# Ruthenium Complexes Containing Thiobenzamide Act as Potent and Selective Anti-*Trypanosoma cruzi* Agents through Apoptotic Cell Death

**DOI:** 10.1021/acsinfecdis.5c00864

**Published:** 2026-01-08

**Authors:** Maria Vitória Gomes das Neves, Isabela Santos Cezar, Edivaldo dos Santos Rodrigues, Felipe Cardoso Teixeira Bomfim, Ricardo da Silva Duarte, Claudia Valeria Campos de Souza, Vinícius Pinto Costa Rocha, Denise Santos de Sá, Osvaldo Andrade Santos-Filho, Carlos Daniel Silva da Silva, Milena Botelho Pereira Soares, Cássio Santana Meira

**Affiliations:** † Gonçalo Moniz Institute, 42509 Oswaldo Cruz Foundation, FIOCRUZ, Salvador, Bahia 40296-710, Brazil; ‡ Institute of Innovation in Advanced Health Systems (ISI SAS), SENAI/CIMATEC University, Salvador, Bahia 41650-010, Brazil; § Laboratory of Molecular Modeling and Computational Structural Biology, Walter Mors Natural Products Research Institute, Health Sciences Center, 28125 Federal University of Rio de Janeiro, Rio de Janeiro, Rio de Janeiro 21941-599, Brazil; ∥ Department of Chemistry, 169704 Federal Institute of Bahia, IFBA, Salvador, Bahia 40301-015, Brazil

**Keywords:** Chagas disease, molecular docking, ruthenium complexes, thiobenzamide, *T. cruzi*, tryponathione reductase

## Abstract

Chagas disease remains a significant global health concern, with current therapies limited to benznidazole and nifurtimox, which have adverse effects and show reduced efficacy in the chronic phase. This study investigated ruthenium complexes with or without thiobenzamide (Tbz). FOR0012A and FOR0212A, both containing Tbz, showed potent trypanocidal activity, with IC_50_ values of 0.13 and 0.09 μM for trypomastigotes, and 1.8 and 0.32 μM for amastigotes. Electron microscopy revealed shrinkage, blebbing, and severe mitochondrial/kinetoplast damage, indicating apoptosis-like cell death, as confirmed by flow cytometry. Docking studies demonstrated strong binding to trypanothione reductase, suggesting oxidative stress induction, further supported by mitochondrial superoxide production and membrane depolarization. In a murine model, FOR0212A (20 mg/kg) reduced parasitemia by 50.2% during the acute phase without any toxicity. These findings identify FOR0212A as a promising therapeutic candidate for Chagas disease, acting via oxidative stress and apoptosis-like mechanisms in *T. cruzi*.

## Graphical abstract

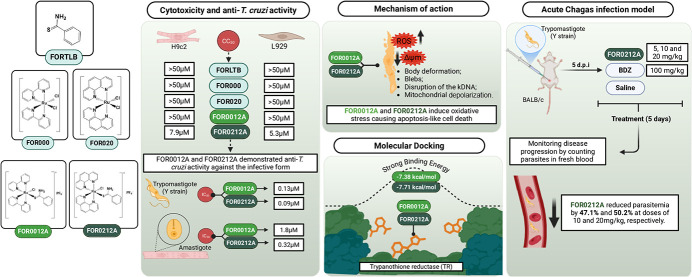

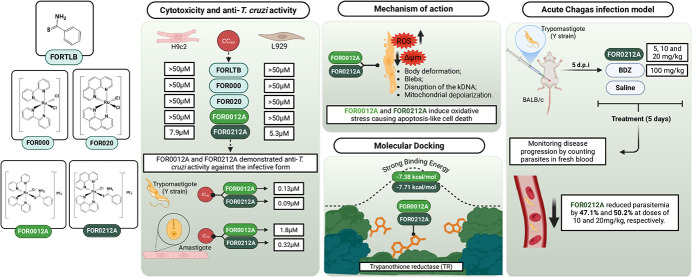

## Introduction

Chagas disease (CD), characterized by its silent progression, is caused by the hemoflagellate protozoan *Trypanosoma cruzi* and ranks among the most prevalent Neglected Tropical Diseases (NTDs) worldwide.^1^ It is estimated that between 6 and 7 million people are affected globally, with the highest incidence in Latin America, where the disease is endemic in 21 countries.^2^ Annually, approximately 30 000 new cases and 12 000 deaths are reported, and around 70 million people in the Americas live in at-risk areas, making them susceptible to infection.^2,3^

Despite its significant public health burden, therapeutic options for CD remain limited. Currently, only two nitroheterocyclic compounds are available for the etiological treatment: benznidazole (BNZ) and nifurtimox (NFX).^4,5^ While both are effective during the acute phase of the disease, their efficacy in the chronic phase is considerably reduced. Moreover, they are associated with numerous adverse effects, such as skin reactions, gastrointestinal disturbances, and central/peripheral nervous system toxicity, often leading to high treatment dropout rates.^4–7^

In response to this scenario, new therapeutic strategies are being explored, including drug repositioning, alternative treatment regimens with BNZ or NFX, and combination therapies.^8^ Additionally, novel compounds are being developed to target essential biological pathways in the parasite’s lifecycle, such as enzymes involved in sterol biosynthesis and specific metabolic routes.^8^ Among these enzymes, trypanothione reductase stands out as it plays a critical role in the survival of *Trypanosomatidae* family parasites by maintaining redox homeostasis in their metabolism.^9,10^

In this context, metal-based compounds have been explored as therapeutic alternatives since the 16th century.^11^ Among them, ruthenium complexes have emerged as promising agents for parasitic diseases due to their kinetic behavior, which is similar to cisplatin, one of the most well-established metal-based drugs.^12^ Ruthenium complexes also exhibit lower toxicity and can access multiple oxidation states.^13^ Their biological activity, reactivity, solubility, and stability are directly influenced by the nature of their ligands.^12–14^

Ruthenium complexes containing polypyridyl ligands, such as 2,2’-bipyridine and 1,10-phenanthroline, have been extensively studied for their antiparasitic properties. These ligands provide enhanced complex stability and facilitate interactions with key biological targets, including DNA and parasite mitochondria, as well as promoting cellular uptake.^15,16^ Such complexes have shown efficacy against protozoans like *Leishmania* spp. and *T. cruzi*, inducing mitochondrial damage, apoptosis-like processes, and significant morphological alterations.^17,18^ Concurrently, thiobenzamides, members of the thioamide class, have emerged as promising bioactive ligands, with reported antiparasitic, antitubercular, antioxidant, antiulcerogenic, and antitumoral activities.^19^ In light of this, the present study evaluated the anti-*T. cruzi* activity of novel ruthenium complexes containing 2,2’-bipyridine, 1,10-phenanthroline, or thiobenzamide ligands using *in silico*, *in vitro,* and *in vivo* approaches.

## Results

### Evaluation of the Cytotoxic Potential and Anti-*Trypanosoma cruzi* Activity of Ruthenium­(II) Complexes

The cytotoxicity of Ru­(II) complexes was initially assessed in H9c2 (rat cardiomyocytes) and L929 (mouse fibroblasts) cell lines using the AlamarBlue assay. Among the tested compounds, only complex FOR0212A exhibited cytotoxicity at the tested concentrations, with CC_50_ values of 5.3 μM for L929 and 7.9 μM for H9c2. In comparison, the positive control doxorubicin showed CC_50_ values of 0.5 and 1.6 μM for L929 and H9c2, respectively (Table 1).

**Table 1:** Antiparasitic and Cytotoxicity Activities of Ruthenium Complexes^c^

	Trypomastigotes	Amastigotes	L929		H9c2	
Compounds	IC_50_ ± SD (μM)^a^	IC_50_ ± SD (μM)^b^	CC_50_ ± SD (μM)^b^	SI	CC_50_ ± SD (μM)^b^	SI
FORLTB	16 ± 1.4	-	>50	>3.1	>50	>3.1
FOR000	>50	-	>50	-	>50	-
FOR020	>50	-	>50	-	>50	-
FOR0012A	0.13 ± 0.0	1.8 ± 0.3	>50	>385	>50	>385
FOR0212A	0.09 ± 0.0	0.32 ± 0.1	5.3 ± 0.5	58.9	7.9 ± 1.0	87.8
DOXO	-	-	0.5 ± 0.1	-	1.6 ± 0.2	-
BDZ	12.5 ± 0.6	2.5 ± 1.0	>50	>4	>50	>4

a Determined 24 h after incubation with compounds.

b Determined 72 h after incubation with compounds. Values were calculated using concentrations in triplicate, and three independent experiments were performed.

c IC50, inhibitory concentration at 50%; CC50, cytotoxic concentration at 50%; SD, standard deviation; BDZ, benznidazole; DOXO, doxorubicin; L929, fibroblast-like from mice; H9c2, neonatal rat cardiomyocytes; SI, Selectivity Index.

Subsequently, the trypanocidal activity of the metal complexes was evaluated against trypomastigote forms of *T. cruzi*. Complexes FOR000 and FOR020 showed no anti-*T. cruzi* activity at the tested concentrations. However, the free ligand Tbz had an IC_50_ value of 16 μM. Notably, complexes FOR0012A and FOR0212A displayed potent trypanocidal activity, with IC_50_ values of 0.13 and 0.09 μM, respectively, both more effective than the reference drug benznidazole (IC_50_ = 12.5 μM) (Table 1).

Based on the CC_50_ and IC_50_ values, the selectivity index (SI) was calculated to determine the compounds’ preference for the parasite over mammalian cells. FOR0012A and FOR0212A exhibited SI values greater than 50, indicating high selectivity (Table 1). Consequently, only these two complexes were selected for further analyses.

To further characterize the anti-*T. cruzi* effect, the selected complexes were evaluated against the intracellular forms of the parasite. Complexes FOR0012A and FOR0212A exhibited IC_50_ values of 1.8 and 0.3 μM, respectively (Table 1). Under the same experimental conditions, BDZ showed an IC_50_ value of 2.5 μM (Table 1).

### Electron Microscopy Analysis

*T. cruzi* trypomastigotes were treated with the Ru­(II) complexes FOR0012A and FOR0212A for 24 h to assess ultrastructural alterations using scanning electron microscopy (Figure 1). Untreated parasites displayed a preserved morphology (Figure 2A). However, treatment with FOR0012A at 0.065 and 0.13 μM (corresponding to IC_50_/2 and IC_50_, respectively) resulted in body shrinkage and contortion of the trypomastigotes (Figure 2B and C). At 0.26 μM (2× IC_50_), FOR0012A caused marked body deformation and the appearance of cytoplasmic membrane projections (blebs) (Figure 2D). Similarly, treatment with FOR0212A at 0.09 μM (IC_50_) induced body contortion and deformation (Figure 2E), while the 0.18 μM concentration (2× IC_50_) led to shrinkage, body twisting, and membrane blebbing (Figure 2F).

**Figure 1:**
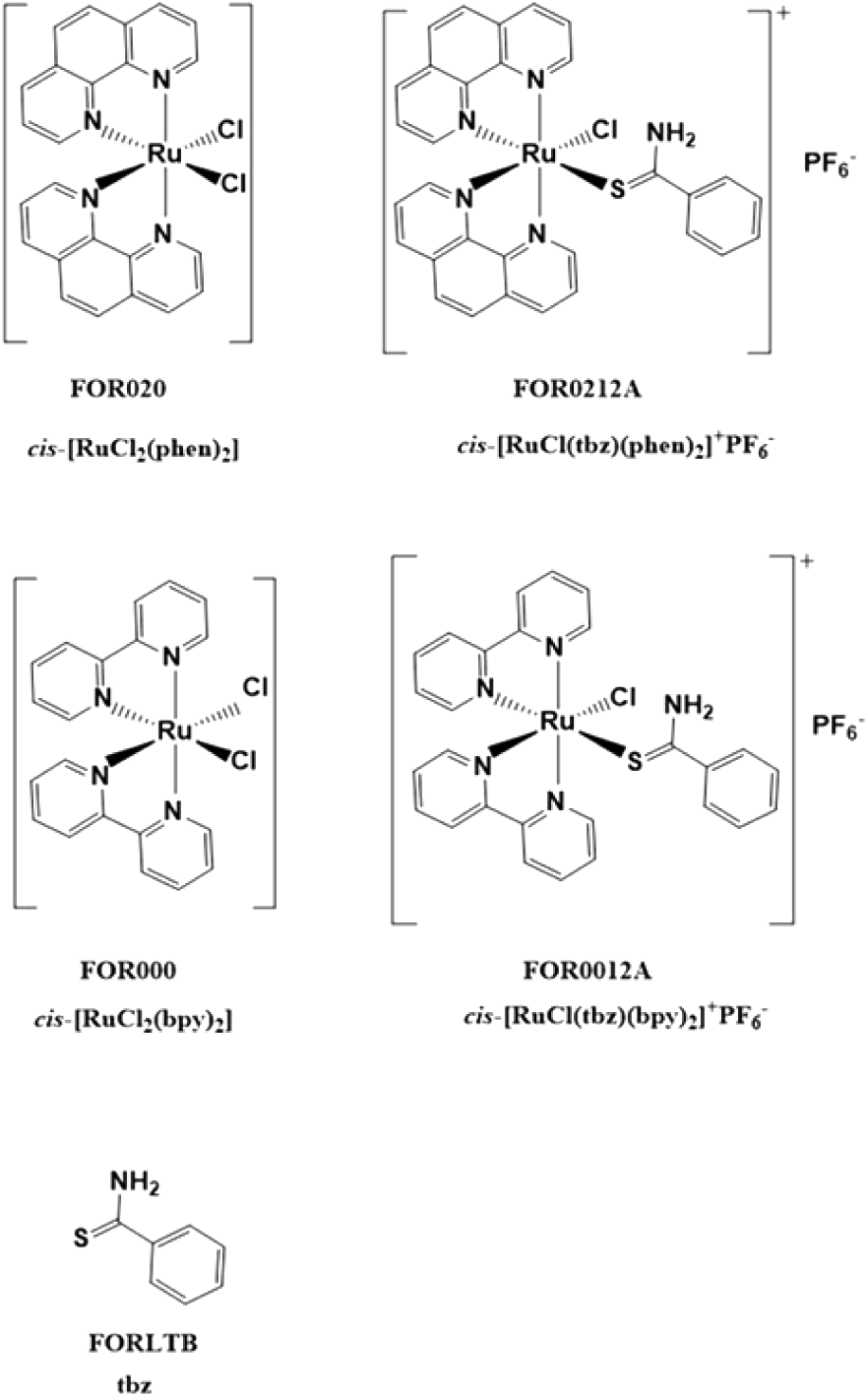
Structure of the ruthenium complexes FOR020, FOR0212A, FOR000, FOR0012A, and FORLTB.

**Figure 2:**
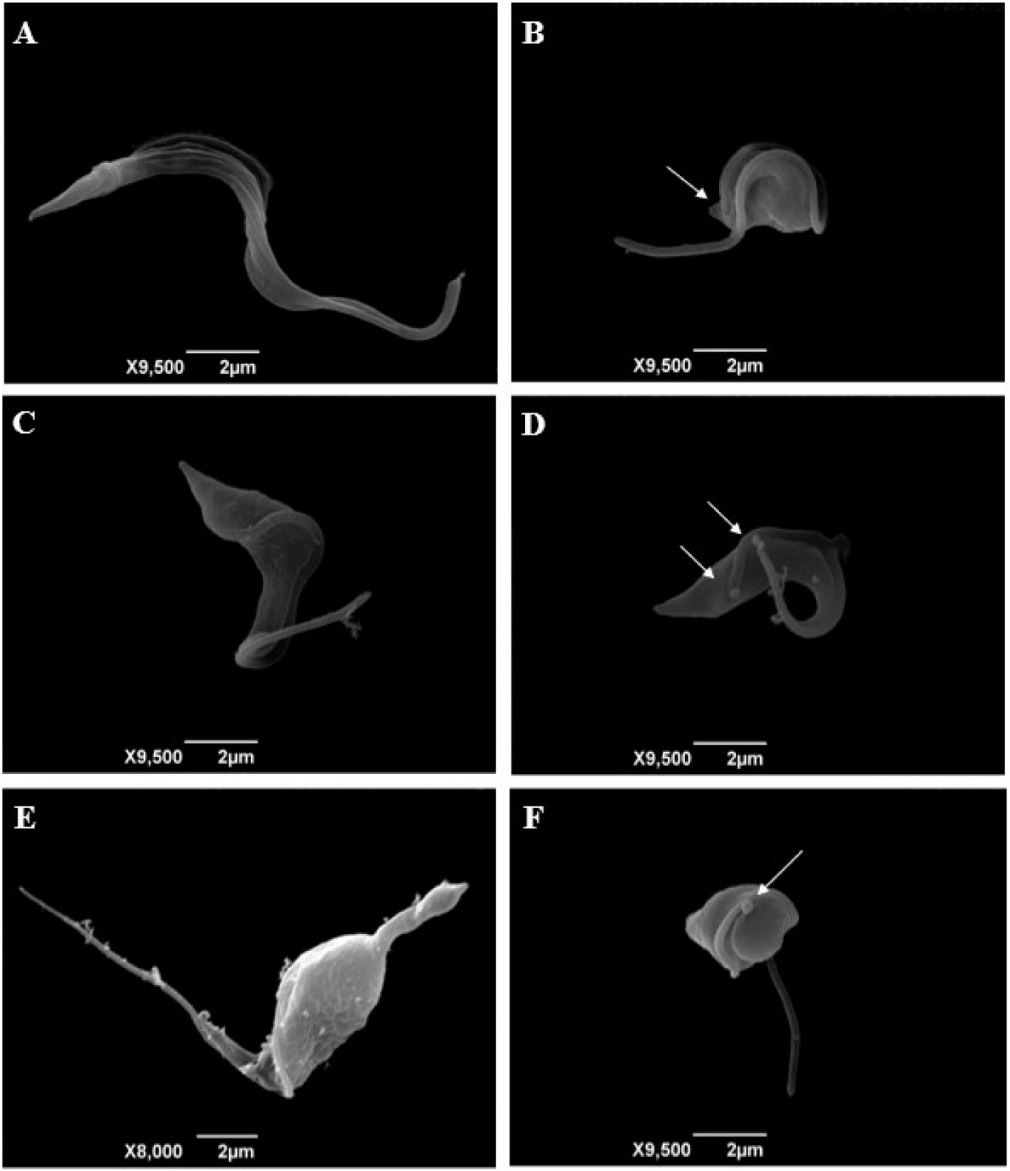
Ultrastructural alterations observed by scanning electron microscopy. *T. cruzi* trypomastigotes were treated with the Ru­(II) complexes FOR0012A (IC_50_/2, IC_50_, and 2 × IC_50_, respectively) and FOR0212A (IC_50_ and 2 × IC_50_, respectively) for 24 h and observed using scanning electron microscopy. (A) Untreated trypomastigotes, showing typical morphology. (B) Trypomastigotes treated with 0.065 μM FOR0012A displayed shrinkage, body deformation, and membrane discontinuity. (C) Treatment with 0.13 μM FOR0012A led to body contortion and deformation. (D) At 0.26 μM FOR0012A, parasites exhibited shrinkage, deformation, and bleb formation on the membrane. (E) Trypomastigotes treated with 0.09 μM FOR0212A showed body deformation and contortion. (F) Treatment with 0.18 μM FOR0212A resulted in shrinkage, body contortion, and membrane blebbing. Morphological changes are indicated by white arrows.

Next, intracellular alterations in the *T. cruzi* trypomastigote forms after treatment with ruthenium complexes were evaluated by transmission electron microscopy. We observed that untreated parasites exhibited an intact intracellular organization with preserved nuclear morphology and intact organelles (Figure 3A). However, trypomastigotes treated with the FOR0012A and FOR0212A complexes showed notable alterations in their organelles. Treatment promoted a loss of electron density in the nuclear region due to genetic material loss, as well as discontinuity of the nuclear envelope (Figure 3B–F). In addition, the FOR0012A and FOR0212A complexes at 0.065 and 0.045 μM, respectively, induced mitochondrial degeneration in trypomastigotes (Figure 3B and E). Parasites treated with FOR0012A and FOR0212A at 0.13 and 0.09 μM, respectively, exhibited kinetoplast disorganization, leading to an electron-lucent appearance due to the loss of kDNA network integrity (Figure 3C and F). Furthermore, treatment with FOR0212A at 0.09 μM induced structural modifications in the endoplasmic reticulum of the parasites (Figure 3E).

**Figure 3:**
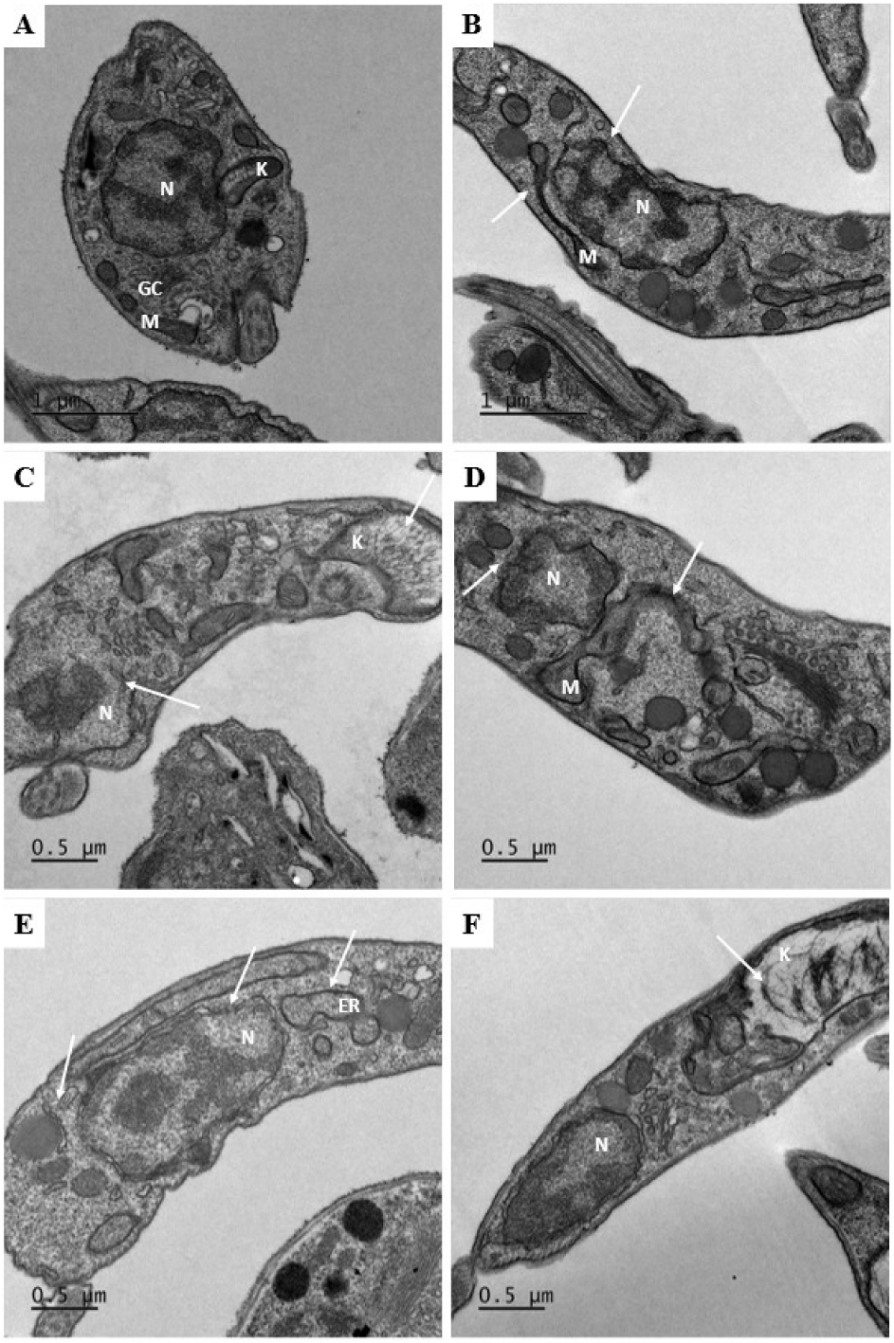
Ultrastructural alterations were observed by transmission electron microscopy. *T. cruzi* trypomastigote forms were treated with the complexes FOR0012A (IC_50_/2, IC_50_, and 2 × IC_50_, respectively) and FOR0212A (IC_50_/2 and IC_50_, respectively) for 24 h. (A) Untreated trypomastigotes showing a delimited nucleus (N) and organelles with normal morphological aspects (mitochondrion: M, kinetoplast: K, and Golgi complex; GC). (B) Trypomastigotes treated with 0.065 μM FOR0012A. (C) Trypomastigotes treated with 0.13 μM FOR0012A. (D) Trypomastigotes treated with 0.26 μM FOR0012A. (E) Trypomastigotes treated with 0.045 μM FOR0212A. (F) Trypomastigotes treated with 0.09 μM FOR0212A. Treatment with the complexes FOR0012A and FOR0212A resulted in the alteration of the endoplasmic reticulum profile (E), mitochondrial degeneration (B and E), enlargement of the kinetoplast accompanied by disruption of the kDNA network (C and F), and an electron-lucent nuclear appearance indicating the loss of genetic material (B–F). All alterations are indicated by white arrows.

### Investigation of the Trypanocidal Pathways of Ru­(II) Complexes

To elucidate the mechanism of action of the FOR0012A and FOR0212A complexes, a new series of experiments was conducted using flow cytometry. Initially, the cell death pattern induced by the complexes in *T. cruzi* trypomastigotes was assessed through annexin V and propidium iodide (PI) staining. The results revealed that both complexes significantly increased (*p < 0.05) the proportion of annexin V-positive parasites, suggesting the induction of apoptosis (Figure 4A and B).

**Figure 4:**
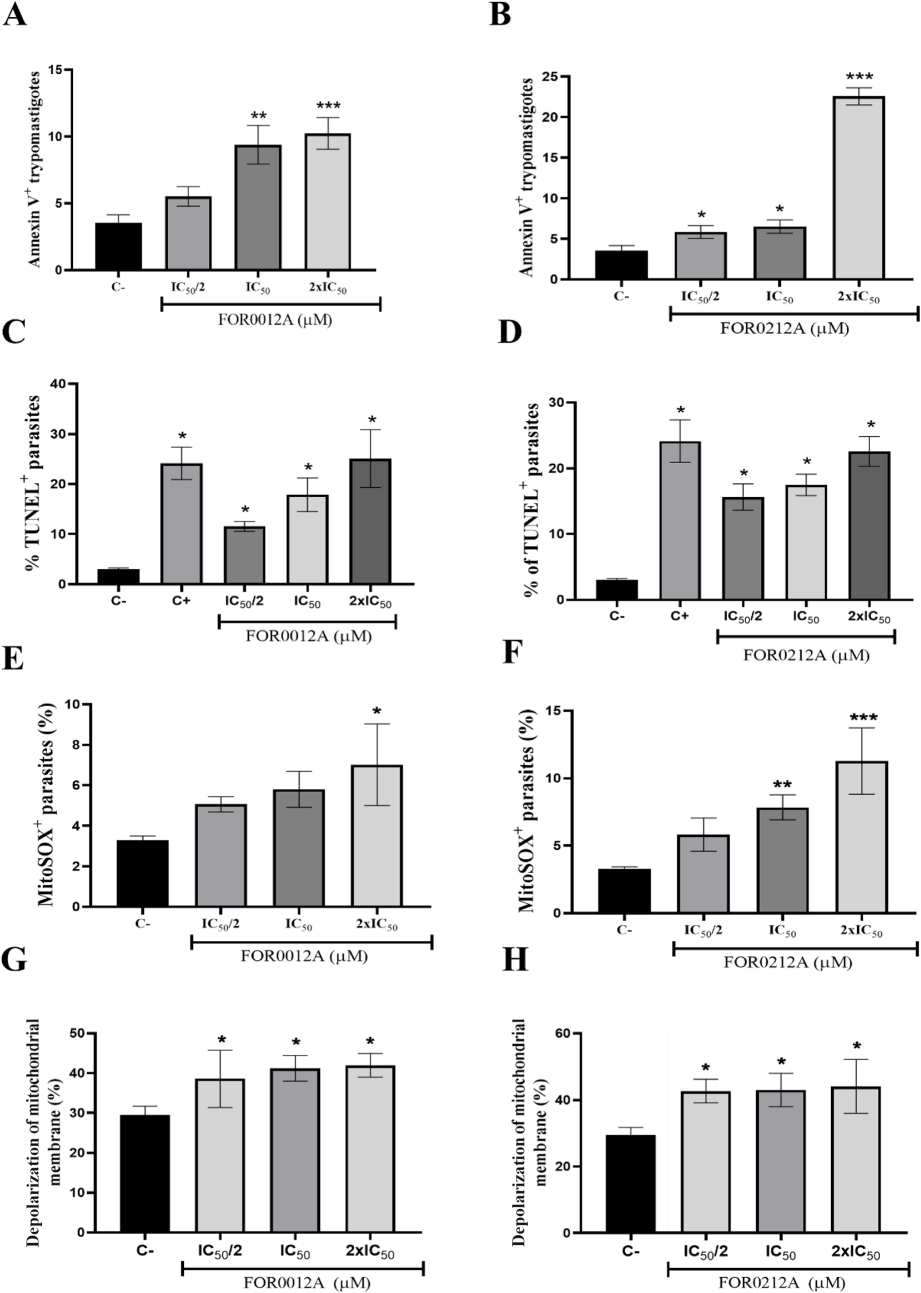
Ruthenium complexes FOR0012A and FOR0212A induce cell death through apoptosis and mitochondrial oxidative stress in *T. cruzi* trypomastigotes. Parasites were treated with FOR0012A and FOR0212A (IC_50_/2, IC_50_, and 2 × IC_50_) for 3 or 24 h and stained with annexin V and PI (A-–B), TUNEL (C–D), mitoSOX (E-–F), or rhodamine 123 (G-–H) for flow cytometry analysis. (A-–B) Apoptosis after 24 h; (C–D) DNA fragmentation after 24 h; (E-–F) Mitochondrial ROS after 3 h; (G-–H) Mitochondrial membrane potential after 24 h. Data represent mean ± SD of triplicates. *p < 0.05 vs control.

Subsequently, to further investigate apoptosis as the mechanism of cell death, *T. cruzi* trypomastigotes were treated with the complexes and analyzed by using the TUNEL assay. The results showed a significant increase (*p* < 0.05) in DNA fragmentation in a concentration-dependent manner, supporting the hypothesis of apoptosis-mediated cell death (Figure 4C and D). Parasites treated with the FOR0012A complex exhibited 17.9% and 25% TUNEL-positive cells at IC_50_ and 2× IC_50_ concentrations, respectively. Similarly, treatment with FOR0212A resulted in 17.4% and 22.5% DNA labeling at the corresponding concentrations.

Given that mitochondria are major sources of superoxide production and play a central role in apoptosis initiation [13], mitochondrial superoxide levels were quantified using the MitoSOX reagent following treatment. The results showed a significant increase (*p < 0.05) in superoxide production in a concentration-dependent manner, indicating the induction of mitochondrial oxidative stress (Figure 4E and F). Additionally, mitochondrial membrane depolarization was evaluated using Rhodamine 123. Treatment with FOR0012A and FOR0212A resulted in a significant (*p < 0.05) increase in mitochondrial membrane depolarization (Figure 4G and H).

### Evaluation of the Interaction of Ru­(II) Complexes with the Trypanothione Reductase

In order to understand the possible interactions and differences in the activity of FOR0212A and FOR0012A as inhibitors of *T. cruzi* trypanothione reductase (TR), molecular docking simulations were carried out by using the MetalDock software. In addition, calculations of three descriptors of reactivity, molecular electrostatic potential (MEP), electron affinity (EA), and hardness at the B3LYP/def2-TZVP level of theory were performed. These latter calculation might help to give a better distinction of the metallic compound’s activity.

From the best binding poses predicted by the docking simulations, the contacts made by FOR0012A and FOR0212A with the protein residues were calculated on the BIOVIA Discovery Studio software, and the corresponding interactions are depicted in Figure 5A,B, respectively. This analysis shows that FOR0012A interacts directly with the machinery catalytic residues, forming a π–sulfur and π–π stacked shape with His461, a π–anion and salt bridge with Glu466, and a π–alkyl with Cys53. On the other hand, FOR0212A only interacts via van der Waals forces with these residues. It does, preferentially, π-type interactions with Tryp22, Tyr111, Leu18, and Ileu339: π–π T-shape, π–π stacked shape, π–lone pair, and π–alkyl. It also forms a salt bridge with Glu19 and carbon–hydrogen bonds with Tyr111 and Glu19. Finally, it is worth mentioning that FOR0012A interacts by van der Waals forces with the chain A residues: Phe396, Leu399, Pro462, Thr463, and Ser464; and the chain B residues: Ser15, Glu19, Val59, and Thr335; while FOR0212A interacts with the residues: Glu466, Gly459, His461, Ser470, and Arg472 on chain A; and with the chain B residues: Gly14, Ser15, Gly50, Cys53, Val54, Val59, Met114, and Pro336. Other relevant interactions and corresponding distances are shown in Figure 5.

**Figure 5:**
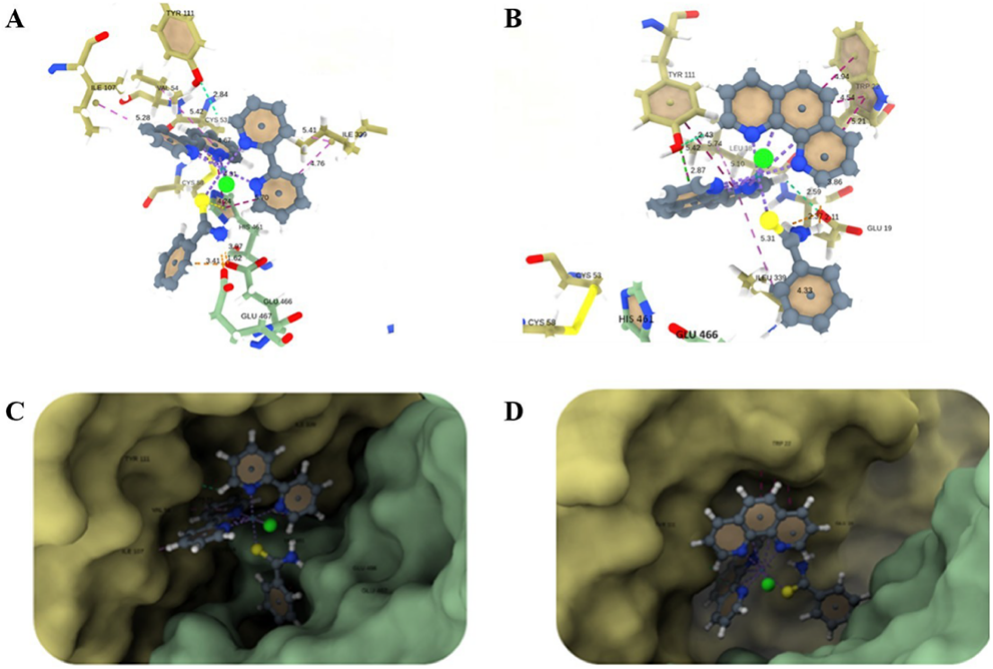
Figure shows the best binding poses predicted by MetalDock for the metallic complexes on the TR enzyme catalytic site (PDB code ID: 1BZL), alongside the direct intermolecular interactions. (A) and (B) show the interactions and corresponding distances of FOR0012A and FOR0212A on the catalytic site, respectively; (C) and (D) show FOR0012A and FOR0212A and the enzyme surface. The metallic complexes are in Ball-and-Stick representation, and residues are shown as Sticks. The metallic complex atoms are colored as slate gray (carbon), blue (nitrogen), yellow (sulfur), green (chlorine), and teal (ruthenium); on the enzyme, the oxygen atoms are in red and carbon atoms are in dark sea green color for chain A residues and dark khaki for chain B residues. The dashed line in purple color represents the ligand-metal bonds, and the others represent the intermolecular interactions: π–π T-shaped and π–π stacked (medium violet red color); π–alkyl (orchid color); π–sulfur (yellow color); carbon–hydrogen bond (medium spring green color); and π–lone pair (lime color). The distances are in Å, and the labels are in black color.

By the MEP shown in Figure 6A and B, we see that FOR0212A has deep regions of charge depletions, called π-holes.^20–22^ These π-holes have a significant impact on the strength of π-type interactions, especially those with anions and lone pairs.^23–25^ Figure 6C and D shows the HOMO and LUMO orbitals alongside the EA and hardness values for both compounds. The lower hardness and higher EA value of FOR0212A mean that this cation is softer and more acidic than FOR0012A; thus, the hydrophobic and polarizable residues of the TR binding pocket might stabilize this compound. Finally, the more symmetrically delocalized positive charge on FOR0012A—seen on the MEP—indicates a less directionality in binding the site, i.e., there will be less repulsion between the negative partial charge of Cl coordinated to the metallic complexes and the carboxyl groups of Glu19, Glu466, and Glu467. The fact that FOR0012A binds deeper into the binding site, Figure 5B and C, might be a reflection of this.

**Figure 6:**
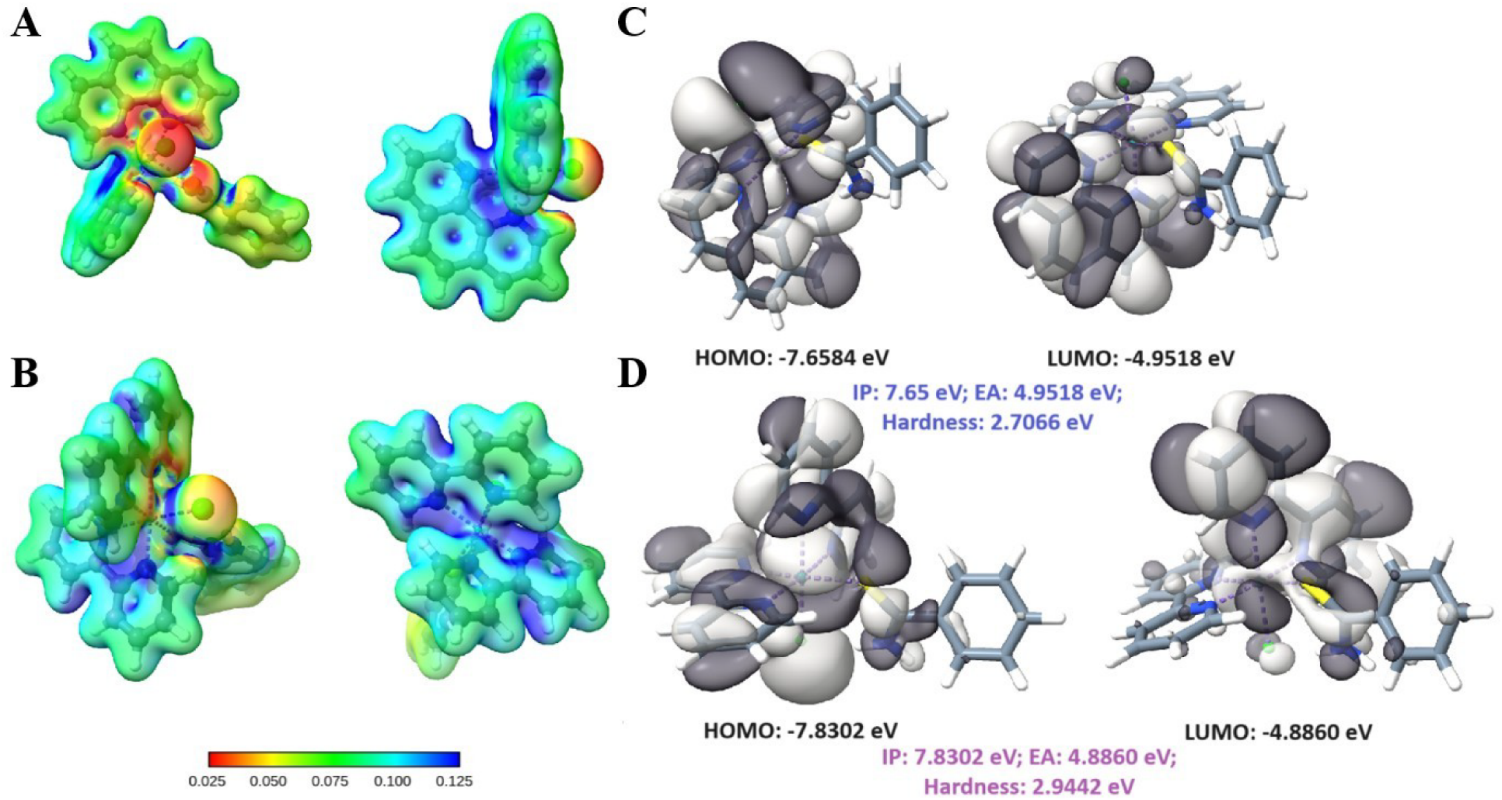
(A) FOR0212A molecular electrostatic potential (MEP); (B) FOR0012A MEP; (C) HOMO and LUMO and their energy values, along with the chemical descriptors—IP, EA, and hardness—in blue font for the FOR0212A complex; (D) HOMO and LUMO, along with their energy values, and the chemical descriptors—IP, EA, and hardness—in pink font for the FOR0012A complex.

Finally, the free energy of binding predicted by MetalDock shows that FOR0212A (−7.71 kcal/mol) is more favorable for binding to the catalytic site of TR compared with FOR0012A (−7.38 kcal/mol). However, as mentioned in the last paragraph, FOR0012A has better steric recognition by the binding site and deeper interaction. This does not mean, though, that FOR0212A does not bind closer to the machinery catalytic site (Cys53, Cys58, His461, and Glu466). From the docking results, other clusters that contained this binding mode were observed, but with lower free energy of binding. Therefore, the docking results, together with the interaction analyses discussed above, lead to the conclusion that FOR0212A might have better affinity for the TR binding site and FOR0012A better binding specificity.

### Evaluation of the Toxic and Trypanocidal Potential of FOR0212A in a Murine Model

Following the evaluation of the cytotoxic and trypanocidal potential of the Ru­(II) complexes in *in vitro* models, only the FOR0212A complex was selected for *in vivo* studies due to its greater potency (IC_50_ = 0.09 μM) compared with the FOR0012A complex. The selection of FOR0212A was also supported by its superior solubility in saline solution (data not shown), which facilitated its administration and enhanced its efficacy in the assays.

Initially, an acute toxicity assay was conducted using female BALB/c mice. Animals received a single oral dose of FOR0212A at different doses (5, 10, and 20 mg/kg) and were monitored for 14 days for behavioral, physical, and body weight changes, which were recorded on days 0, 7, and 14. No behavioral alterations, morphological abnormalities, or significant changes in body weight were observed, as shown in Tables 2 and 3.

**Table 2:** Effect of FOR0212A on the Behavioral and General Appearance of Female BALB/c Mice^a^

	Observations			
Behavior and general appearance	Vehicle	FOR0212A(5 mg/kg)	FOR0212A(10 mg/kg)	FOR0212A(20 mg/kg)
**Changes in the eyes**	No changes	No changes	No changes	No changes
**Changes in the fur**	No changes	No changes	No changes	No changes
**Changes in the skin**	No changes	No changes	No changes	No changes
**Coma**	Absent	Absent	Absent	Absent
**Convulsions**	Absent	Absent	Absent	Absent
**Diarrhea**	Absent	Absent	Absent	Absent
**Lethargy**	Absent	Absent	Absent	Absent
**Salivation**	Absent	Absent	Absent	Absent
**Sleep**	Usual	Usual	Usual	Usual
**Tremors**	Absent	Absent	Absent	Absent

a The animals were observed daily for 14 days.

**Table 3:** Body Weight of BALB/c Mice Treated with the Compound FOR0212A^a^

Days	Vehicle	FOR0212A(5 mg/kg)	FOR0212A(10 mg/kg)	FOR0212A(20 mg/kg)
0	22.0 (±1.6)	23.7 (±1.9)	22.6 (±1.2)	23.6 (±1.8)
7	23.9 (±1.3)	24.5 (±1.2)	23.1 (±1.4)	24.5 (±1.7)
14	25.0 (±1.9)	26.2 (±1.4)	24.6 (±1.4)	25.7 (±2.1)

a Values represent the mean ± standard deviation of five animals per group.

Subsequently, an acute *T. cruzi* infection model was employed using female BALB/c mice. Animals were infected intraperitoneally with 10^4^ trypomastigotes, and on the fifth day postinfection, they were treated orally for 5 consecutive days with FOR0212A (5, 10, and 20 mg/kg), benznidazole (100 mg/kg), or vehicle solution (saline containing 5% DMSO). Parasitemia levels were monitored on days 5, 8, 10, and 12 postinfection. Treatment with FOR0212A at doses of 10 and 20 mg/kg significantly reduced parasitemia (47.1% and 50.2%, respectively; *p < 0.05) compared to the vehicle-treated group (Figure 7). Under the same conditions, benznidazole treatment resulted in a parasitemia reduction greater than 99.5% (*p < 0.05).

**Figure 7:**
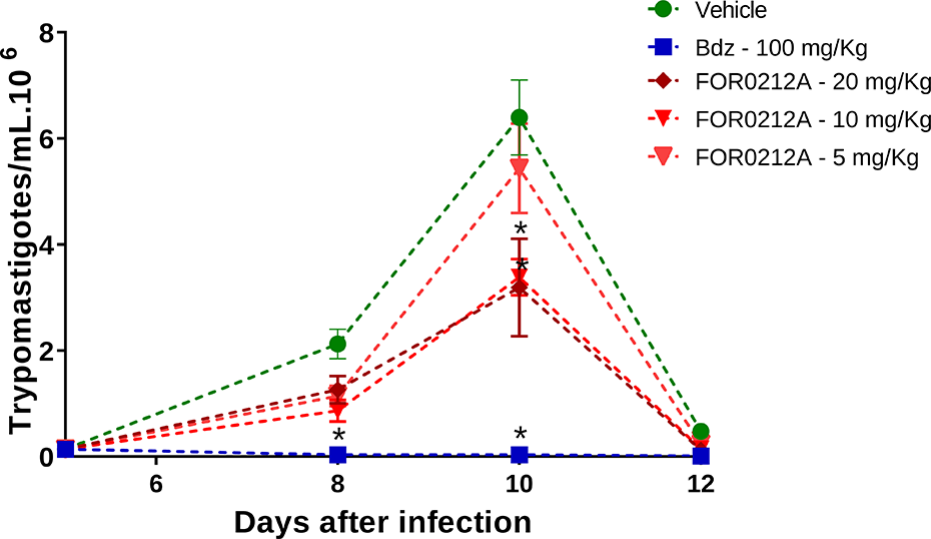
Parasitemia in BALB/c mice infected with *T. cruzi* and treated with the FOR0212A complex. Female BALB/c mice were infected with 10^4^ trypomastigotes of *T. cruzi* (Y strain). Five days postinfection, animals were orally treated with the FOR0212A complex (5, 10, and 20 mg/kg) or benznidazole (100 mg/kg) for 5 consecutive days. Parasitemia was monitored by counting trypomastigotes in fresh blood samples. Data represent the mean ± standard deviation of six mice per group from one of two independent experiments. *p < 0.05 compared to the vehicle-treated group.

## Discussion

The discovery of new drugs for the treatment of Chagas disease is an urgent priority, as current therapeutic options are limited to benznidazole or nifurtimox, both of which are associated with significant adverse effects.^4,6^ In this context, metal-based compounds have emerged as promising candidates against *T. cruzi* due to their structural diversity and geometric flexibility, which allow interaction with multiple parasite-specific biological targets.^26–28^ These compounds may act through selective membrane accumulation, DNA damage, or inhibition of essential enzymes required for parasite survival.^28–30^

In the present study, we evaluated the anti-*T. cruzi* potential of novel ruthenium complexes through *in vitro* assays on different parasite stages (trypomastigotes and amastigotes). Complexes lacking the thiobenzamide ligand (FOR000 and FOR020) did not inhibit trypomastigote viability. In contrast, the thiobenzamide-containing complexes FOR0012A and FOR0212A significantly reduced both trypomastigote viability and amastigote proliferation. Among them, FOR0212A showed lower IC_50_ values than FOR0012A, likely due to the extended aromatic rings and higher redox potential of the *o*-phenanthroline ligand in comparison to the 2,2’-bipyridine in FOR0012A.^19^

Previous studies from our group reported ultrastructural alterations in *T. cruzi* trypomastigotes treated with ruthenium complexes, such as parasite shrinkage and contortion.^17,18^ Moreover, literature reports indicate that treatment with such metal complexes in *Leishmania amazonensis* promastigotes and *Trypanosoma brucei* leads to mitochondrial swelling or degeneration, kinetoplast disorganization, and cytoplasmic content loss, which confer an electron-lucent appearance to the parasites.^31–33^

Apoptosis-like cell death in trypanosomatids is phenotypically characterized by DNA fragmentation, phosphatidylserine exposure on the plasma membrane, mitochondrial membrane potential (ΔΨm) loss, cytochrome c release, and increased production of reactive oxygen species (ROS).^31,34,35^ In this study, treatment with FOR0012A and FOR0212A promoted phosphatidylserine externalization, DNA fragmentation, increased mitochondrial superoxide levels, and alterations in the ΔΨm. The increase in ROS may be associated with ΔΨm loss and Ca^2+^ influx imbalance, triggering oxidative stress, which is typically associated with early apoptotic events in trypanosomatids.^36–39^ These findings align with the morphological changes observed by electron microscopy and support the induction of apoptosis-like cell death by FOR0012A and FOR0212A.

Under oxidative stress conditions, trypanosomatids rely on an antioxidant defense system centered on the enzyme trypanothione reductase (TryR), which catalyzes the NADPH-dependent reduction of trypanothione disulfide (TS_2_) to trypanothione (T­(SH)_2_).^10,40^ Based on this, we performed molecular docking analyses to explore the interaction of the ruthenium complexes with TryR’s active site. FOR0212A showed a higher binding affinity, while FOR0012A exhibited greater binding specificity. These results are consistent with *in vitro* data, in which FOR0212A was more potent against trypomastigotes, whereas FOR0012A showed greater selectivity. Furthermore, these findings support the hypothesis that both complexes promote oxidative stress through TryR inhibition.

We therefore selected the compound FOR0212A to evaluate in a mouse model of acute *Trypanosoma cruzi* infection due to its promising *in vitro* profile since it showed superior trypanocidal potency and solubility (data not shown). Treatment with FOR0212A at 10 or 20 mg/kg significantly reduced parasitemia, demonstrating a strong trypanocidal effect, although without surpassing the efficacy of benznidazole under comparable conditions. This outcome is consistent with previous studies reporting similar levels of activity for other ruthenium-based complexes. For example, the 1H-indazole-containing complex FOR0E2, administered at 20 mg/kg, achieved a 36.7% reduction in parasitemia, though with demonstrated mitochondrial disruption and DNA fragmentation in parasites, likely due to the inhibition of trypanothione reductase.^18^ Other NO-donor ruthenium complexes, similar to complex 3, were previously shown to present potent *in vivo* activity, reducing parasitemia and increasing survival rates via nitric oxide release that induced autophagic and necrotic parasite death.^17^ These findings highlight FOR0212A as a promising therapeutic candidate whose performance aligns with or exceeds that of structurally distinct, bioactive ruthenium complexes targeting *T. cruzi* through diverse mechanisms.

## Conclusion

In this study, complexes containing the thiobenzamide moiety (FOR0012A and FOR0212A) exhibited potent and selective trypanocidal activity against distinct evolutionary forms of the parasite, including trypomastigotes and amastigotes. In contrast, the complexes lacking the thiobenzamide moiety showed no activity, indicating that this functional group is essential for the antiparasitic effect. This is also reinforced by the activity of thiobenzamide against trypomastigotes being slightly greater than that of benznidazole. Regarding the mechanism of action, both FOR0012A and FOR0212A interacted with the active site of the enzyme trypanothione reductase (TryR), inducing oxidative stress followed by apoptosis-like cell death. Furthermore, FOR0212A demonstrated significant efficacy in reducing parasitemia in a murine model of acute *T. cruzi* infection, without evidence of toxicity in the treated groups.

## Methods

### Drugs

The Ru­(II) complexes *cis*-[RuCl­(tbz)­(phen)_2_]^+^PF6^–^ (FOR0212A), *cis*-[RuCl­(tbz)­(bpy)_2_]^+^PF6^–^ (FOR0012A), *cis*-[RuCl_2_(bpy)_2_] (FOR000), *cis*-[RuCl_2_(phen)_2_] (FOR020), and Thiobenzamide (FORLTB) (Figure 1) were synthesized as previously described.^19,41^ Doxorubicin hydrochloride, used as a positive control in cytotoxicity assays, was obtained from the IMA S.A.I.C. Laboratory (Buenos Aires, Argentina). Benznidazole, used as the reference drug, was acquired from the Pharmaceutical Laboratory of the State of Pernambuco (LAFEPE) (Recife, PE, Brazil).

In all *in vitro* biological activity assays, the compounds were first solubilized in dimethyl sulfoxide (DMSO; Êxodo Científica, São Paulo, SP) and subsequently in Dulbecco’s Modified Eagle’s Medium (DMEM; Life Technologies, GIBCO-BRL, Gaithersburg, MD), ensuring that the final DMSO concentration did not exceed 0.1% in the tests. For *in vivo* assays, the most active complex was first solubilized in 5% DMSO and then in a 95% saline solution.

### Parasites

Trypomastigote forms of *T. cruzi* (Y strain) were obtained from the supernatant of previously infected *Rhesus* monkey kidney (LLC-MK2) cell cultures. The infected cells were maintained in DMEM medium, supplemented with 10% fetal bovine serum (FBS; Gibco) and 1% Pen-Strep (Gibco), in a humidified incubator at 37 °C with 5% CO_2_. Cell culture passages were performed every 7 days.

### Animals

Male and female BALB/c mice (18–20 g) were housed in sterilized cages under controlled environmental conditions, receiving a balanced rodent diet and water *ad libitum* at the Gonçalo Moniz Research Center (Oswaldo Cruz Foundation, Bahia, Brazil). All experiments were conducted in accordance with ethical guidelines and were approved by the local Animal Ethics Committee (protocol number 016/2023).

### Assessment of Cytotoxicity to H9c2 and L929 Cells

The cells were plated in 96-well plates at a density of 5 × 10^3^ cells/well in DMEM medium supplemented with 10% FBS and 1% Pen-Strep and incubated for 24 h at 37 °C with 5% CO_2_. Subsequently, the complexes were added at different concentrations (50–0.39 μM) and incubated under the same conditions for 72 h. Afterward, 10% AlamarBlue (Invitrogen, Carlsbad, CA) was added, and the plate was incubated again for 4 h. The plate was then read using a spectrophotometer (SpectraMax 190, Molecular Devices, Sunnyvale, CA) at wavelengths of 570 and 600 nm. The CC_50_ values were calculated using data from three independent experiments.

### Antiparasitic Activity

The trypomastigote forms of *T. cruzi* were plated at a density of 4 × 10^5^ parasites per well in 96-well plates and incubated at 37 °C with 5% CO_2_, along with ruthenium complexes at varying concentrations (10–0.07 μM). After 24 h, viable parasites were counted using a Neubauer chamber, based on morphology and motility. The different complex concentrations were used to determine the IC_50_ value. Benznidazole was used as a positive control.

### *T. cruzi*-Infected Cardiomyocytes

H9c2 cells were plated at a density of 5 × 10^3^ cells per well in 96-well plates and incubated at 37 °C with 5% CO_2_. After 24 h, cardiomyocytes were infected with *T. cruzi* trypomastigotes at a density of 5 × 10^4^ parasites per well for 2 h. The medium was then removed, and the cells were washed twice with sterile 1× PBS to eliminate noninternalized parasites. The infected cells were incubated for an additional 24 h under the same conditions.

Next, the ruthenium complexes FOR0012A and FOR0212A were added at varying concentrations (4–0.06 μM) and incubated for 72 h. Benznidazole (10 μM) was included as a positive control. After the incubation period, the medium was removed, and the cells were washed with 1× PBS and fixed with 4% paraformaldehyde. The cardiomyocytes were then stained with DRAQ5 dye (1:1000) (Sigma-Aldrich, St. Louis, MO), and images were acquired using the Cell Insight CX7 Content Analysis Platform (Thermo Fisher Scientific, Waltham, MA) with a 10× objective.

### Investigation by Scanning Electron Microscopy

Trypomastigote forms of *T. cruzi* were plated at a density of 3 × 10^7^ parasites per well in 6-well plates and incubated with the ruthenium complexes FOR0012A and FOR0212A at different concentrations (IC_50_/2, IC_50_, and 2× IC_50_) for 24 h at 37 °C with 5% CO_2_. After incubation, the parasites were centrifuged, washed with saline solution, and fixed for 1 h at room temperature in a solution containing 2% formaldehyde and 2.5% glutaraldehyde (Electron Microscopy Sciences, Hatfield, PA) in 0.1 M cacodylate buffer (pH 7.2). The samples were then washed with 0.1 M cacodylate buffer, adhered to coverslips previously coated with poly-l-lysine (Sigma-Aldrich), and postfixed with osmium tetroxide solution at 1% (OsO_4_; Sigma-Aldrich) containing 0.8% potassium ferrocyanide. Next, the samples underwent a dehydration process in a graded ethanol series (30, 50, 70, 90, and 100%), followed by critical point drying and a gold sputter coating. Finally, the samples were analyzed using a JEOL JSM-6390LV scanning electron microscope at 12 kilovolts (kV).

### Investigation by Transmission Electron Microscopy

Trypomastigote forms of *T. cruzi* were plated at a density of 3 × 10^7^ parasites per well in 6-well plates and treated with the ruthenium complexes FOR0012A and FOR0212A at concentrations of IC_50_/2, IC_50_, and 2× IC_50_ for 24 h in a humidified incubator at 37 °C with 5% CO_2_. After incubation, the parasites were centrifuged, washed with saline solution, and fixed for 24 h at room temperature in the dark with 2% formaldehyde and 2.5% glutaraldehyde in cacodylate buffer (0.1 M; pH 7.4). The samples then underwent a graded dehydration process in acetone (30, 50, 70, 90, and 100%) before being embedded in Polybed resin (Polysciences, Washington, PA). Finally, ultrathin sections were prepared using a Leica UC7 ultramicrotome, collected on 300-mesh copper grids, and contrasted with uranyl acetate and lead citrate. Images were acquired by using a JEOL TEM-1230 transmission electron microscope.

### Investigation of the Type of Cell Death

Trypomastigotes were plated at a density of 2× 10^6^ parasites per well in 24-well plates and treated with the ruthenium complexes FOR0012A and FOR0212A at different concentrations (IC_50_/2, IC_50_, and 2× IC_50_) for 24 h in a humidified incubator at 37 °C and 5% CO_2_. After incubation, the parasites were centrifuged and stained with propidium iodide (PI) and annexin V using the Annexin V (FITC) Apoptosis Detection Kit (BD Biosciences) for 15 min, following the manufacturer’s instructions. Data acquisition was performed by collecting 50 000 events using the BD LSRFortessa flow cytometer, and data analysis was conducted using FlowJo 10 software (FlowJo LLC, Ashland, OR).

### DNA Fragmentation Assessment

Trypomastigote forms were plated at a density of 5 × 10^6^ parasites per well in 24-well plates and treated with the ruthenium complexes FOR0012A and FOR0212A at different concentrations (IC_50_/2, IC_50_, and 2× IC_50_) for 24 h at 37 °C with 5% CO_2_. After incubation, the parasites were centrifuged, washed with saline solution, and fixed with 4% paraformaldehyde (Electron Microscopy Sciences) for 1 h at room temperature. They were then permeabilized with 0.01% Triton on ice for 5 min and stained using the TUNEL kit (Roche Life Science, Switzerland) for 1 h at room temperature in the dark, following the manufacturer’s instructions. Positive controls were incubated with 10 U/mL of DNase I for 10 min. Data acquisition was performed by collecting 50 000 events using the BD LSRFortessa flow cytometer, and data analysis was conducted using FlowJo 10 software.

### Mitochondrial Superoxide Production Analysis

Trypomastigotes were plated at a density of 2 × 10^6^ parasites per well in 24-well plates and incubated with the ruthenium complexes FOR0012A and FOR0212A at different concentrations (IC_50_/2, IC_50_, and 2× IC_50_) for 3 h in a humidified incubator at 37 °C with 5% CO_2_. After incubation, the parasites were washed with saline solution, centrifuged, and stained with MitoSOX Red (Thermo Fisher) for 10 min in the dark, following the manufacturer’s instructions. Data acquisition was performed by collecting 50 000 events using the BD LSRFortessa flow cytometer, and data analysis was conducted using FlowJo 10 software.

### Mitochondrial Membrane Potential Measurement

*T. cruzi* trypomastigotes were plated at a density of 2 × 10^6^ parasites per well in 24-well plates and treated with the ruthenium complexes FOR0012A and FOR0212A at concentrations of IC_50_/2, IC_50_, and 2× IC_50_ for 24 h in a humidified incubator at 37 °C with 5% CO_2_. After incubation, the parasites were centrifuged, washed with saline solution, and stained with Rhodamine 123 (10 μg/mL) (Sigma-Aldrich) for 15 min in the dark, according to the manufacturer’s instructions. Data acquisition was performed by collecting 50 000 events using the BD LSRFortessa flow cytometer, and data analysis was carried out using FlowJo 10 software.

### Molecular Modeling and Docking Simulation

The docking simulations were performed by using the MetalDock software. This program predicts the ligand binding modes by using the AutoDock engine. The main difference, though, is that MetalDock contains parameters specifically for docking metal–organic compounds. Furthermore, MetalDock automates the computation of the partial atomic charges of metal complexes. To achieve this, the user must provide the Cartesian coordinate file of the metal complex for geometry optimization or single-point calculation, provided by one of the following software programs: ORCA, Gaussian, or ADF. Subsequently, the charges are extracted from the single-point calculations using the Charge Model 5 method. For a better understanding of the program workflow and its parametrization procedure, as well as its success and limitations, the following description is provided.

The Cartesian coordinates for the metal complexes (FOR0212A and FOR0012A) were built with the web-based interface for quantum chemistry tools, WebMO, followed by energy optimization using the BP86 GGA density functional implemented in the ORCA program package.^42,43^ These structures were then used as inputs in MetalDock. The B3LYP hybrid density functional was chosen for further geometry optimization and single-point calculations.^44^ All these quantum calculations were done at the low-spin state using def2-TZVP basis set, including Grimme’s dispersion correction D3 with BJ damping and relativistic effects corrected by ZORA.^45^ Finally, the molecular electrostatic potential (MEP) was constructed using the electronic density obtained from the final single-point calculation and the chemical descriptor based on conceptual DFT—the ionization potential (IP), electron affinity (EA), and hardness (η)—was estimated by taking the highest occupied molecular orbital (HOMO) and lowest unoccupied molecular orbital (LUMO) energies (ϵ) and applying the finite difference methods:^46,47^

1 IP=−ϵ(HOMO)

2 EA=−ϵ(LUMO)

3 Hardness=IP−EA

The crystal structure of the *T. cruzi* trypanothione reductase, with a resolution of 2.40 Å and ID code 1BZL16, was obtained from the Protein Data Bank (PDB; https://www.rcsb.org/pdb). According to the workflow of MetalDock and AutoDock, the receptor structure was cleaned by removing water molecules, the FAD cofactor, and the natural substrate (trypanothione, TS2). The enzyme was then protonated at pH 7.4 using the PDB2PQR software. A grid measuring 20 Å × 20 Å × 20 Å, centered at the TS2 position and with Cartesian coordinates defined as *x* = 62.065 Å, *y* = 5.269 Å, and *z* = 3.202 Å, was created for docking simulation purposes. Next, 10 docking simulation runs were performed with the AutoDock4 engine using the default settings for the Lamarckian Genetic Algorithm. From these simulations, the best binding poses were chosen for further analysis.

Finally, the interaction between the metal complexes and the side chains of the amino acids was analyzed with the BIOVIA Discovery Studios software, and graphical treatment of the protein–ligand complex and MEP and HOMO/LUMO orbitals from the metallic compounds was done with the ChimeraX software.

### Acute Toxicity Model

Female BALB/c mice (6–8 weeks old; *n* = 5) were divided into four experimental groups and received a single oral dose of the FOR0212A complex at doses of 5, 10, and 20 mg/kg or the vehicle solution (saline containing 5% DMSO). Following treatment, the animals were monitored for 14 days to assess potential signs of toxicity over a 14-day period. Body weights were measured and recorded on days 0, 7, and 14.

### Acute *T. cruzi* Infection Model

Female BALB/c mice were infected intraperitoneally with 10^4^ *T. cruzi* trypomastigotes, obtained from the supernatant of previously infected LCC-MK2 cell cultures. Five days postinfection, the animals were treated orally once daily for 5 consecutive days with the FOR0212A complex at different doses (5, 10, and 20 mg/kg), benznidazole (100 mg/kg), or the vehicle solution (saline containing 5% DMSO). Parasitemia was assessed macroscopically on days 5, 8, 10, and 12 postinfection.

### Statistical Analysis

The IC_50_ and CC_50_ values were determined using nonlinear regression (curve fitting). The selectivity index (SI) was calculated as the ratio of the CC_50_ value (mammalian cells) to the IC_50_ value (trypomastigote and amastigote forms). Other statistical analyses were performed using one-way analysis of variance (ANOVA), followed by the Newman–Keuls multiple comparison test, with Prism 9.0 software (GraphPad Software, San Diego, CA, USA). A significance level of *p* < 0.05 was considered statistically significant.

## #

Postgraduate Program in Applied Chemistry, State University of Bahia, UNEB, Salvador, Bahia 41150-000, Brazil
